# Supplementary material for: Ambient Mass Spectrometry Imaging Reveals Spatiotemporal Brain Distribution and Neurotransmitter Modulation by 1,8-Cineole: An Epoxy Monoterpene in Mongolian Medicine Sugmel-3
Source: Metabolites. 2025 Sep 22;15(9):631. doi: 10.3390/metabo15090631 (PMC12471961; doi:10.3390/metabo15090631)
Supplement: Supplementary file 1 [file metabolites-15-00631-s001.zip › metabolites-3846315-Supplementary Materials.pdf]

Supplementary Materials

Ambient Mass Spectrometry Imaging Reveals Spatiotemporal Brain Distribution and Neurotransmitter Modulation by 1,8-Cineole: An Epoxy Monoterpene in Mongolian Medicine Sugmel-3

Supplementary methods

LC-MS/MS analysis

LC-MS/MS experiments were conducted on a tandem high-resolution mass spectrometer (TOF™ 6600, AB SCIEX) in both positive ion mode. Brain tissue samples, weighing approximately 100 mg, were homogenized in 400 μL ice water for 60 s using a tissue crusher. A portion of the homogenate (100 μL) was then transferred to a 10 mL glass tube, and a mixture solution of methanol and methyl tert-butyl ether (MeOH: MTBE, 1: 1, V/V) was added. After vortexing at 2500 rpm for 5 min and centrifuging at 4200 rpm for 10 min at 4°C, the supernatant

was carefully transferred to another glass tube. Subsequently, 3 mL of MTBE and 1.2 mL H_2O were added to the remaining residue, followed by vortexing at 2500 rpm for 15 min and centrifuging at 4200 rpm for 10 min at 4°C. The upper organic phase was collected in a separate glass tube, while the lower aqueous phase was transferred to a 2 mL centrifuge tube. After centrifugation and concentration, the highly polar extract from the lower layer was dissolved with 100 μL ACN: H_2O (2:98, V/V), and the weakly polar extract from the upper layer was dissolved with 400 μL IPA: ACN: H_2O (1:1:2, V/V/V). The redissolved samples were vortexed at 2500 rpm for 5 min and then centrifuged at 4°C for 5 min at 12500 rpm (highly polar extract) / 4500 rpm (weakly polar extract). The supernatant was carefully separated and filtered through 96-well plates for LC-MS/MS analysis.

Highly polar extract analysis was performed on a normal phase waters ACQUITY UPLC BEH HILIC column (100 mm × 2.1 mm, 1.7 μm). The mobile phases were H_2O (0.1% formic acid, A) and ACN (B). The mobile phase gradient was set as follows: 98-50% B (0–9 min), 50% B (15 min). The flow rate was set to 0.25 mL/min, with an injection volume of 5 μL, and the column oven temperature was maintained at 35°C.

Weakly polar extract analysis was performed on a reversed-phase waters ACQUITY UPLC^{TM} HSS T3 column (100 mm × 2.1 mm, 1.8 μm). The mobile phases were H_2O (0.1% formic acid+2 mM NH4Ac, A) and ACN: IPA (1:1, v/v, 0.1% formic acid+2 mM NH4Ac, B). The mobile phase gradient was set as follows: 35-50% B (0–1 min), 50-70% B (1–3 min), 70-90% B (3–10 min), 90-100% B (10–15 min), 100% B (15–30 min). The flow rate was set to 0.35 mL/min, with an injection volume of 5 μL, and the column oven temperature was maintained at 45°C.

Mass spectrometry experiments were performed on a Q/TOF-MS system (TOF™ 6600, AB SCIEX, Foster City, USA) equipped with an electrospray ionization (ESI) source. Data was acquired in both positive ion modes. The instrumental parameters were set as follows: ion spray voltage, 5500 / –4500 V; nebulizer gas (GS 1), 30 psi; heater gas (GS 2), 30 psi; curtain gas, 25 psi; ion source temperature, 500°C / 450°C; and declustering potential (DP) 60 / –80 V. the collision energy (CE) was set at 15 eV, 20 eV, 30 eV, 40 ev / –15 eV, –18 eV, –20 eV, –25 eV, –30 eV, –40 eV. The mass ranges were 100–1000 Da for the TOF MS scan and 50–1000 Da for the MS/MS scan.

Supplementary tables and figures

No.	Table/ Figure	Title	Page
1	Table S1	Parameters of AFADESI ion source and Q-Orbitrap mass spectrometer for the MSI analysis of the brain tissue sections.	S-5
2	Figure S1	The microstructure of rat brain.	S-5
3	Figure S2	The relative intensity changes of 1,8-cineole and 2-hydroxy-1,8-cineole in the different brain microregions with the time.	S-6

4	Figure S3	Changes in the relative intensity of 1,8-cineole and the metabolite 2-hydroxy-1,8-cineole in different brain microregions after 5 min, 30 min, 3 h and 6 h of administration.	S-7
5	Figure S4	The relative intensity changes of serotonin in the different brain microregions with the time.	S-8
6	Figure S5	The relative intensity changes of GABA in the different brain microregions with the time.	S-9
7	Figure S6	The relative intensity changes of Glu in the different brain microregions with the time.	S-10
8	Figure S7	The relative intensity changes of Gln in the different brain microregions with the time.	S-11
9	Figure S8	The relative intensity changes of histamine in the different brain microregions with the time.	S-12
10	Figure S9	The relative intensity changes of choline in the different brain microregions with the time.	S-13
11	Figure S10	The relative intensity changes of Ach in the different brain microregions with the time.	S-14
12	Figure S11	The relative intensity changes of Adenosine in the different brain microregions with the time.	S-15
13	Figure S12	The relative intensity changes of melatonin in the different brain microregions with the time.	S-16
14	Figure S13	The relative intensity changes of DA in the different brain microregions with the time.	S-17
15	Figure S14	Relative intensity of NTs in each brain microregion before administration of 1,8-cineole to rats.	S-18
16	Table S2	The detailed information of the detected metabolites by AFADESI-MSI in positive ion mode.	S-18
17	Table S3	Differential Metabolites Detected by Mass Spectrometry Imaging Targeting the Try-5-HT-Mel and Gln-Glu-GABA Metabolic Pathways.	S-19
18	Table S4	Spatial Distribution and Alterations of 5-HT and GABA Pathway Metabolites in Rat Brain 30 min Post 1,8-Cineole Treatment Compared to Control.	S-23

Table S1 Parameters of AFADESI ion source and Q-Orbitrap mass spectrometer for the MSI analysis of the brain tissue sections.

Parameter	Value
Spray Voltage	-7.0 kV
Tube Voltage	-7.0 kV
Spray solvent flow	7 μL/min
Spray gas pressure	0.6 MPa

Extracting gas flow	45 L/min
Spray needle angle	60°
X axis move speed	0.2 mm/s
Y axis step size	0.2 mm
Distance from sprayer to surface	0.6 mm
Distance from sprayer to tube	3 mm
Distance from orifice to tube	10 mm
Spray solvent composition	ACN/H2O (8:2, v/v)

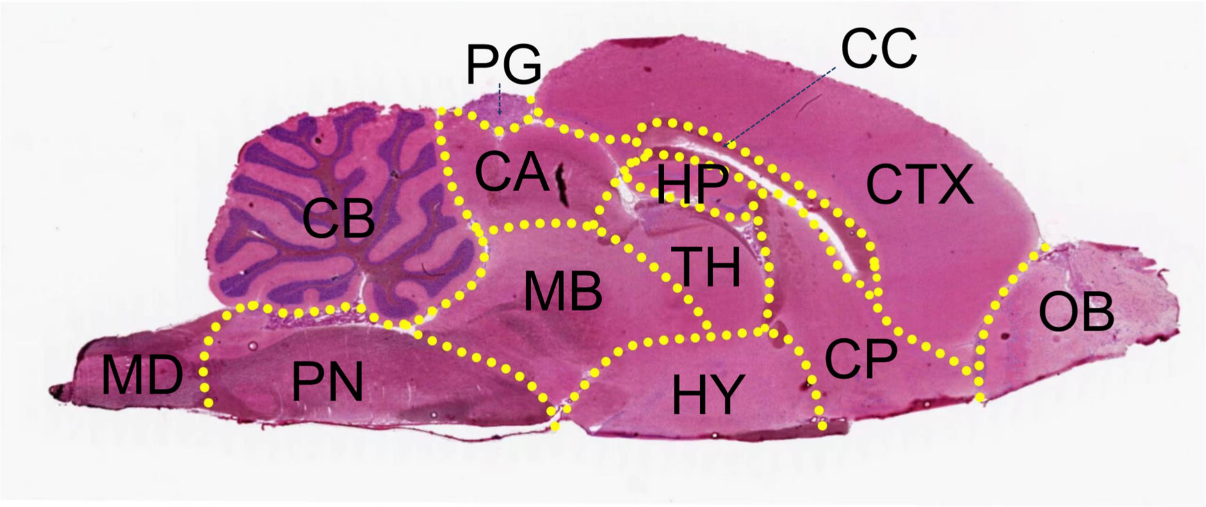

Figure S1 The microstructure of rat brain. PG: pineal gland, CC: corpus callosum, HP: hippocampus, CB: cerebellar, CA: cerebral aqueduct, CTX: cerebral cortex, MB: middle brain, TH: thalamus, MD: medulla, PN: pons; HY: hypothalamus, CP: caudate putamen, OB: olfactory bulb.

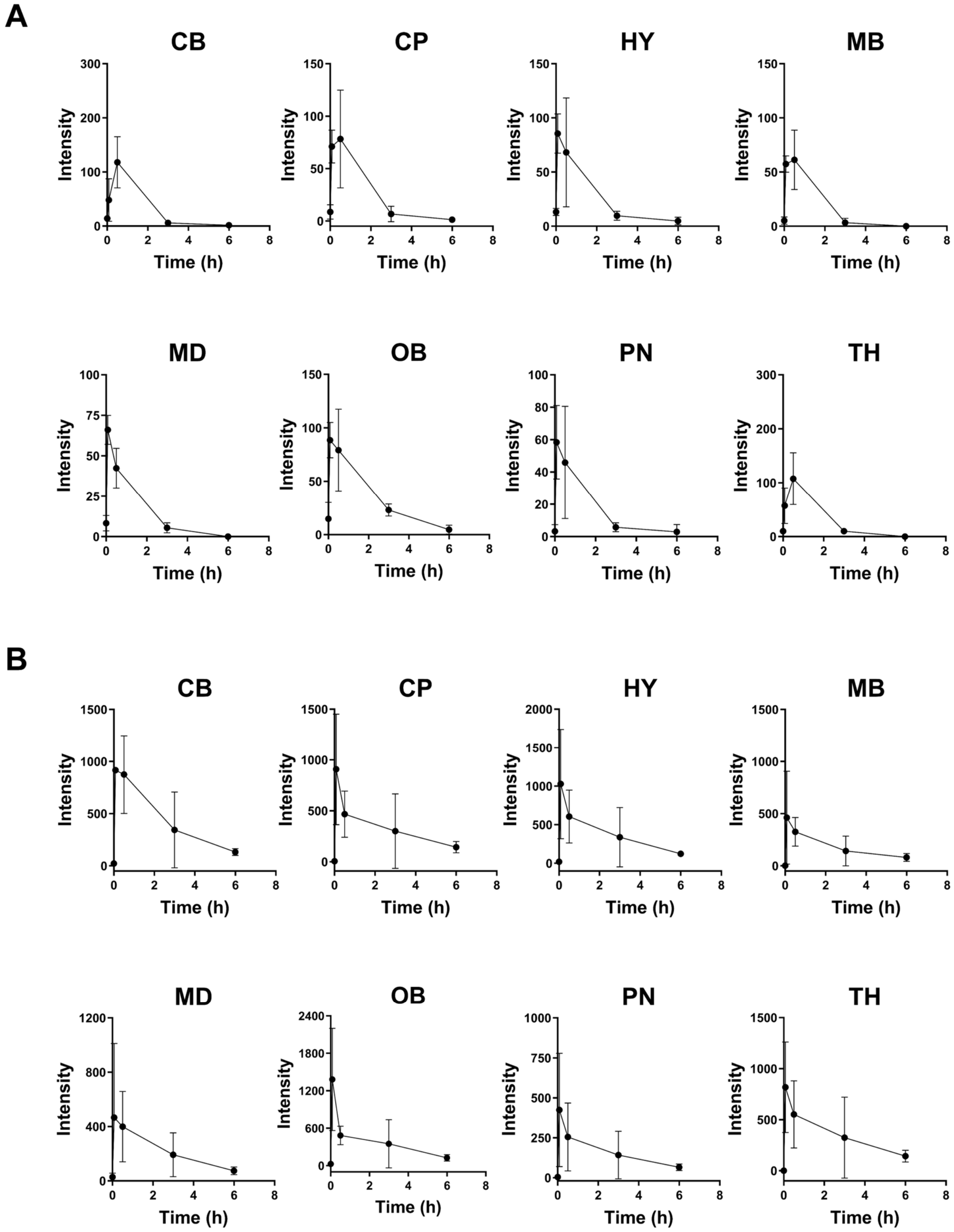

Figure S2 The relative intensity changes of 1,8-Cineole (A) and 2-hydroxy-1,8-Cineole (B) in the different brain microregions with the time. Data are presented as means ± standard deviation (SD), *n* = 3. CB: cerebellar, CP: caudate putamen, HY: hypothalamus: middle brain, MD: medulla, OB: olfactory bulb, PN: pons, TH: thalamus.

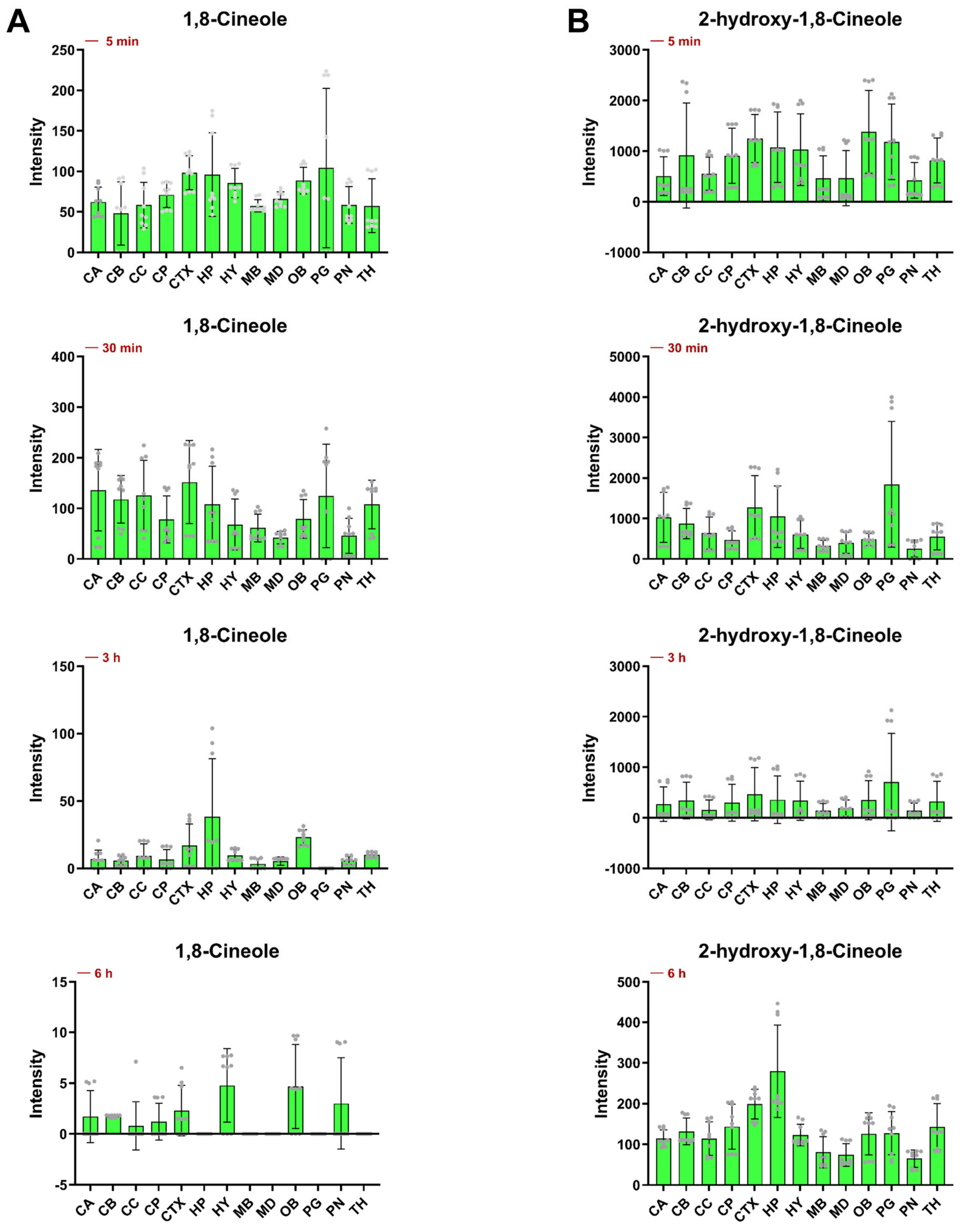

Figure S3 (A, B) Changes in the relative intensity of 1,8-Cineole and the metabolite 2-hydroxy-1,8-Cineole in different brain microregions after 5 min, 30 min, 3 h and 6 h of administration. Data are presented as means ± standard deviation (SD), *n* = 3. PG: pineal gland, CC: corpus callosum, HP: hippocampus, CB: cerebellar, CA: cerebral aqueduct, CTX: cerebral cortex, MB:

middle brain, TH: thalamus, MD: medulla, PN: pons; HY: hypothalamus, CP: caudate putamen, OB: olfactory bulb.

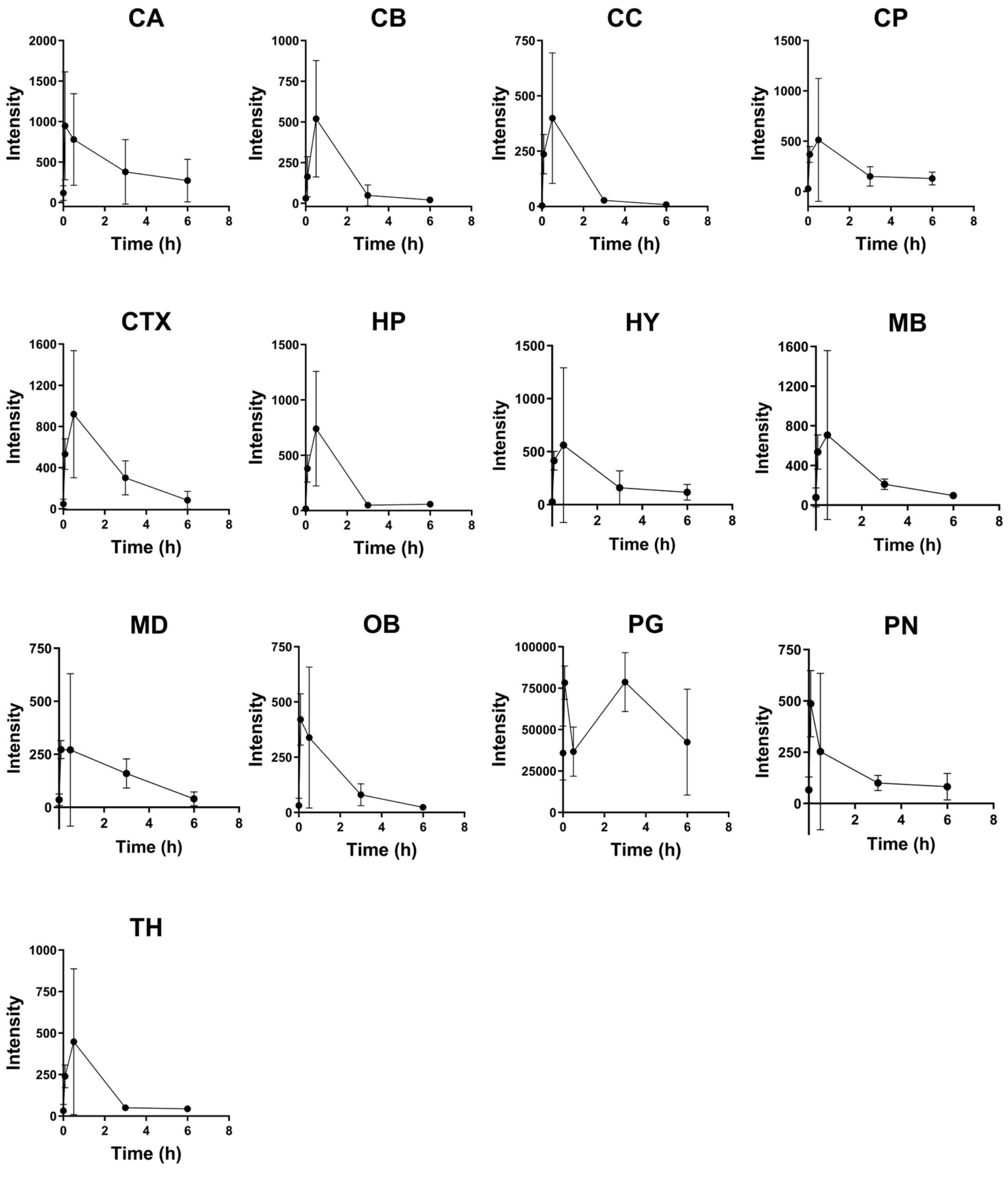

Figure S4 The relative intensity changes of serotonin in the different brain microregions with the time. Data are presented as means ± standard deviation (SD), $n = 3$.

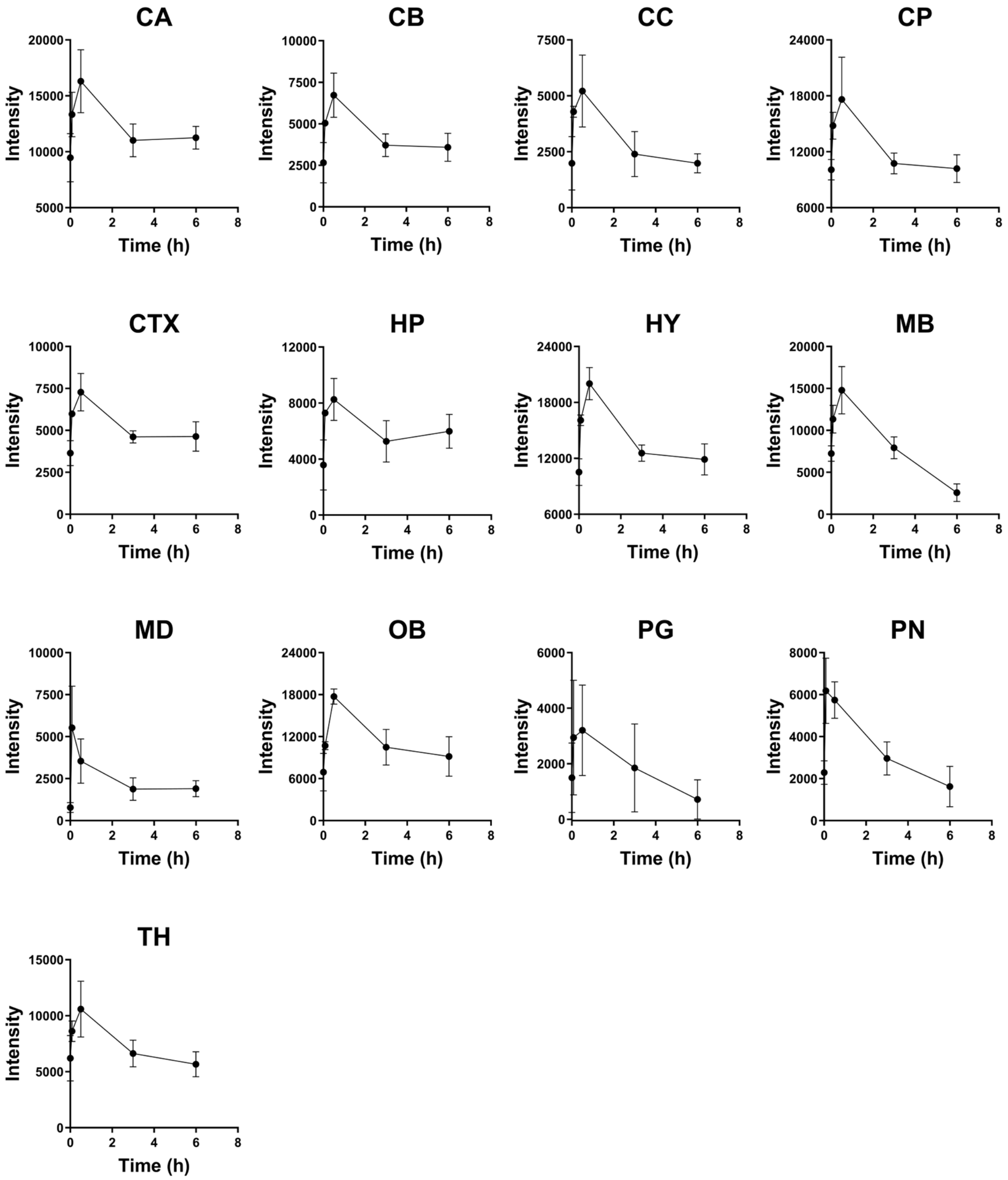

Figure S5 The relative intensity changes of GABA in the different brain microregions with the time. Data are presented as means ± standard deviation (SD), *n* = 3.

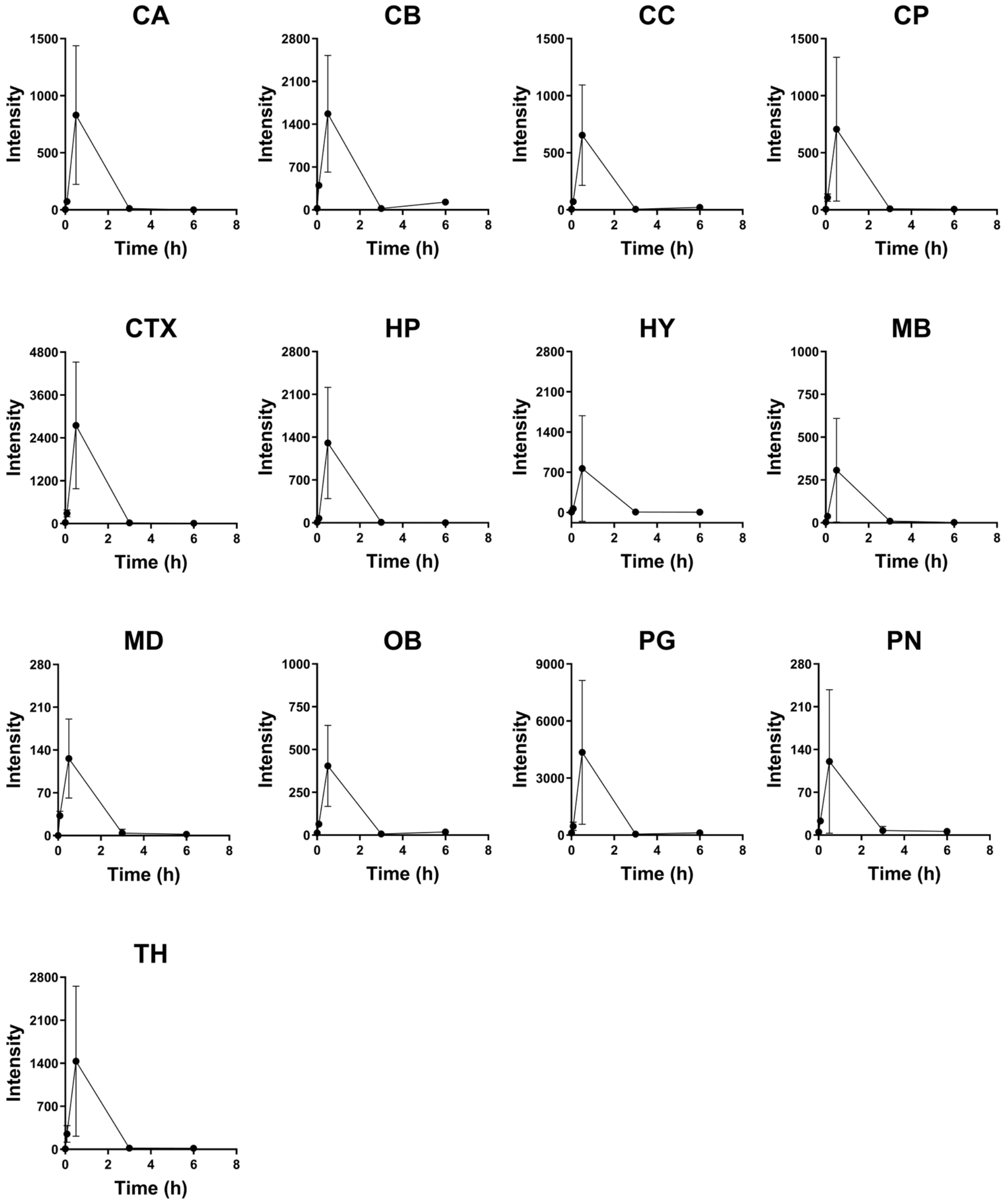

Figure S6 The relative intensity changes of Glu in the different brain microregions with the time. Data are presented as means ± standard deviation (SD), $n = 3$.

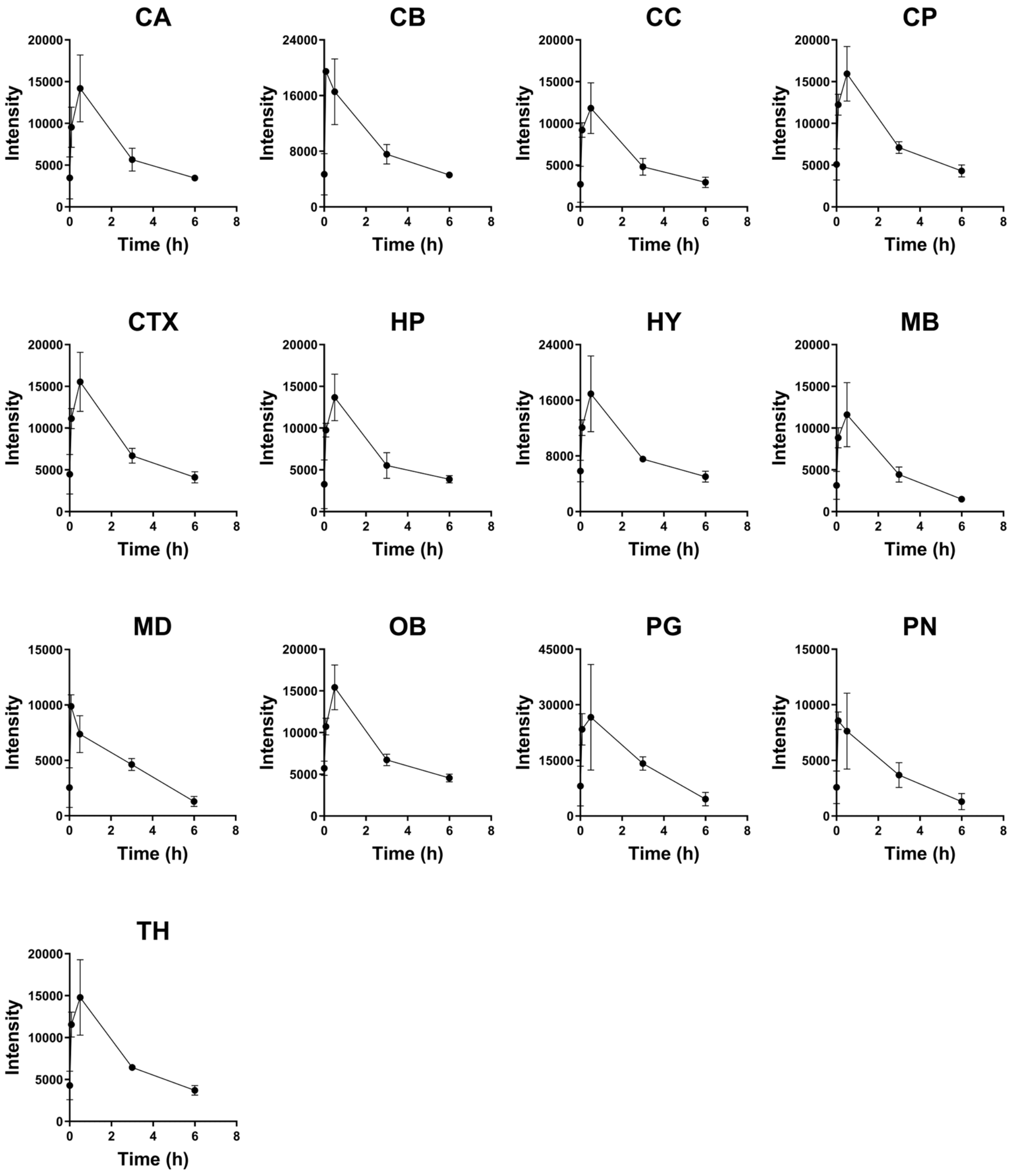

Figure S7 The relative intensity changes of Gln in the different brain microregions with the time. Data are presented as means ± standard deviation (SD), $n = 3$.

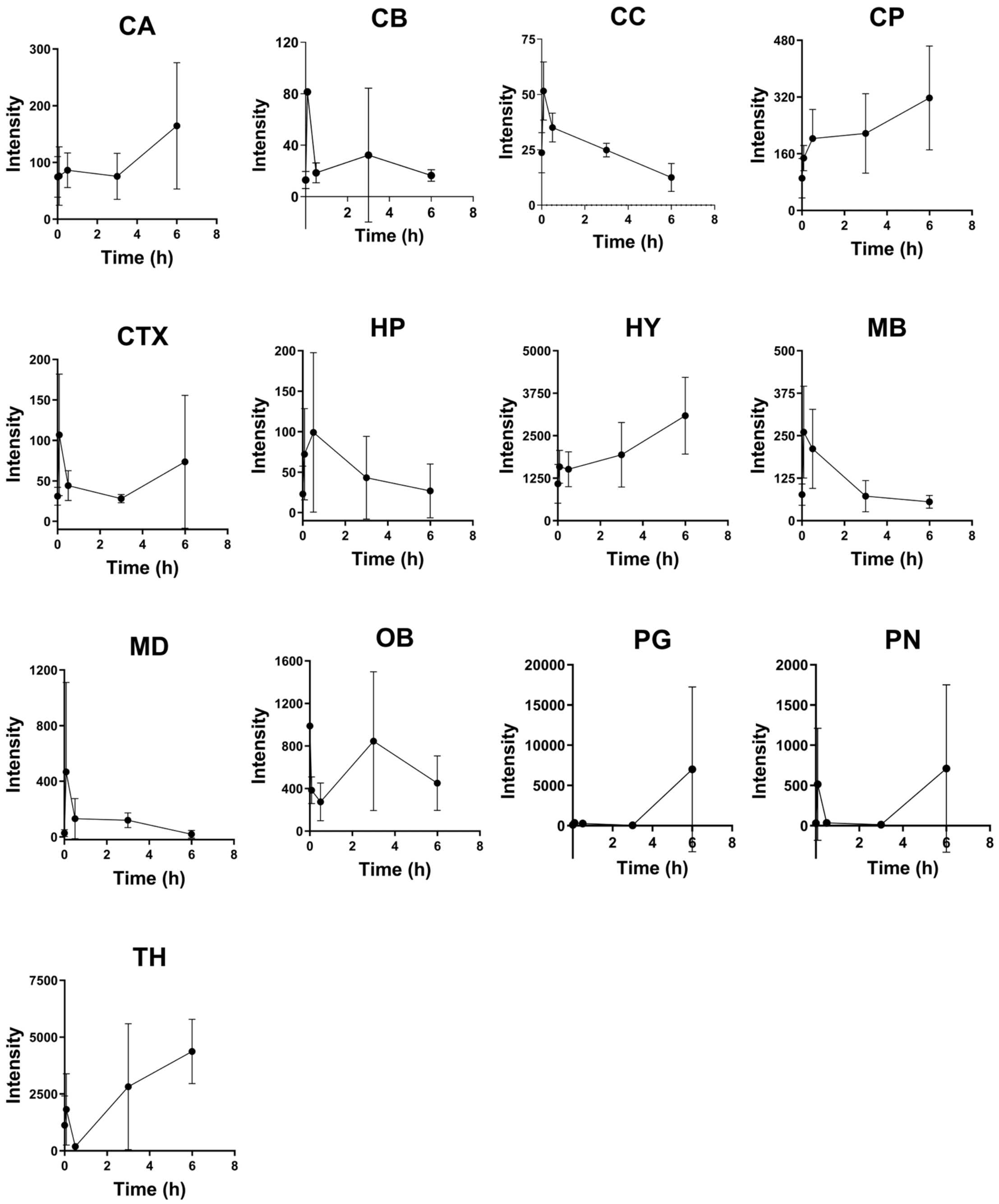

Figure S8 The relative intensity changes of histamine in the different brain microregions with the time. Data are presented as means ± standard deviation (SD), $n = 3$.

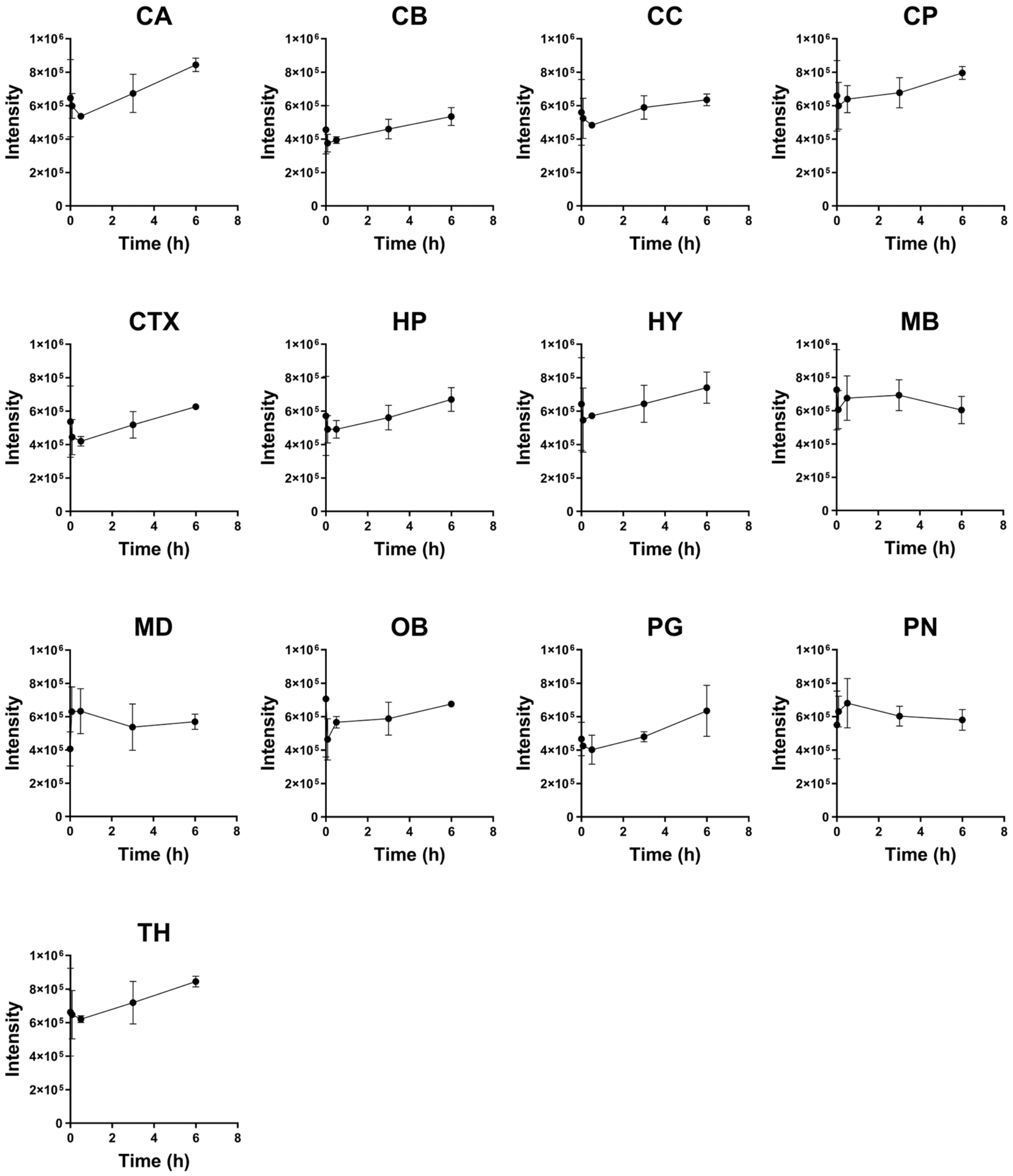

Figure S9 The relative intensity changes of choline in the different brain microregions with the time. Data are presented as means ± standard deviation (SD), n = 3.

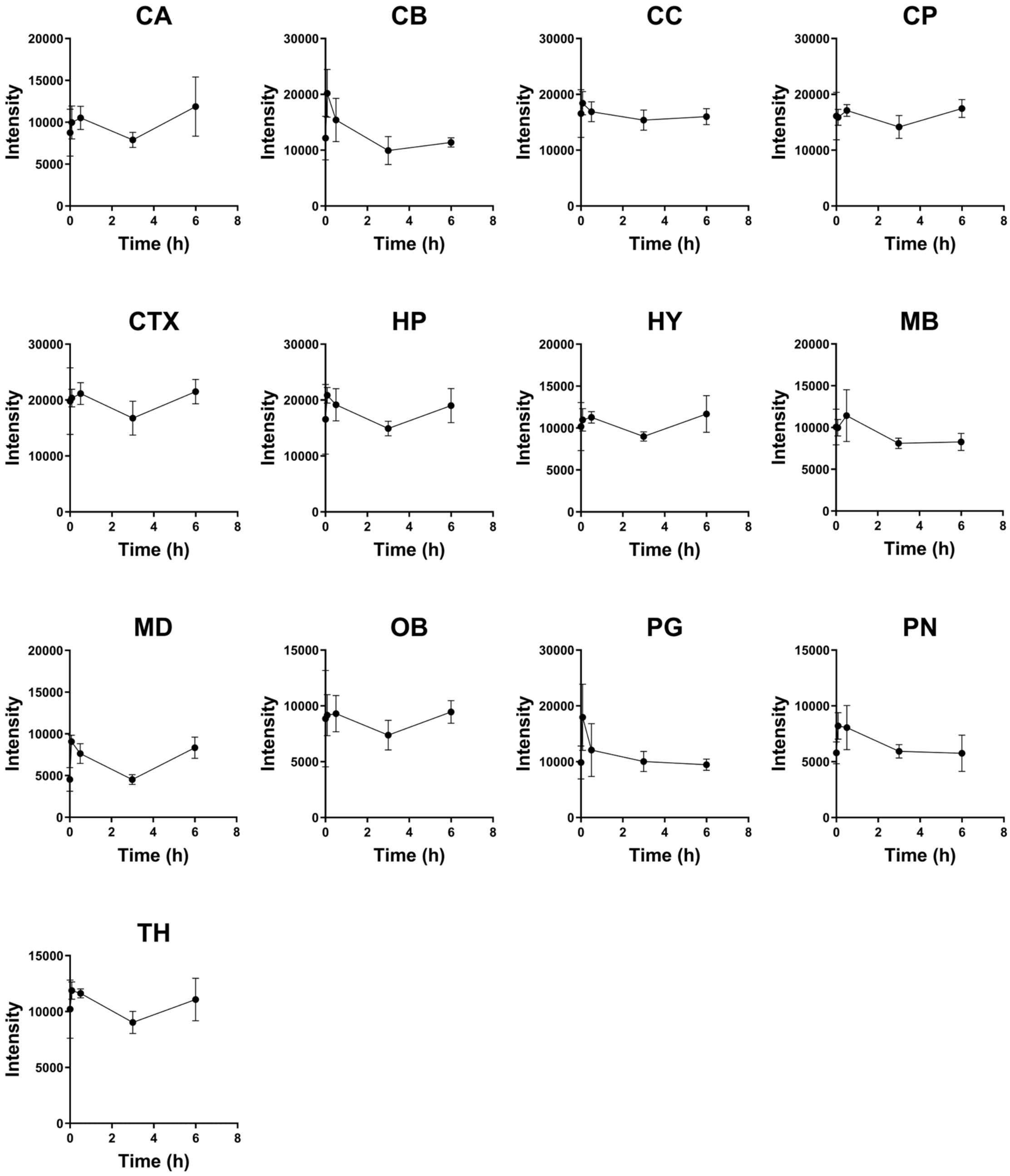

Figure S10 The relative intensity changes of Ach in the different brain microregions with the time. Data are presented as means ± standard deviation (SD), $n = 3$.

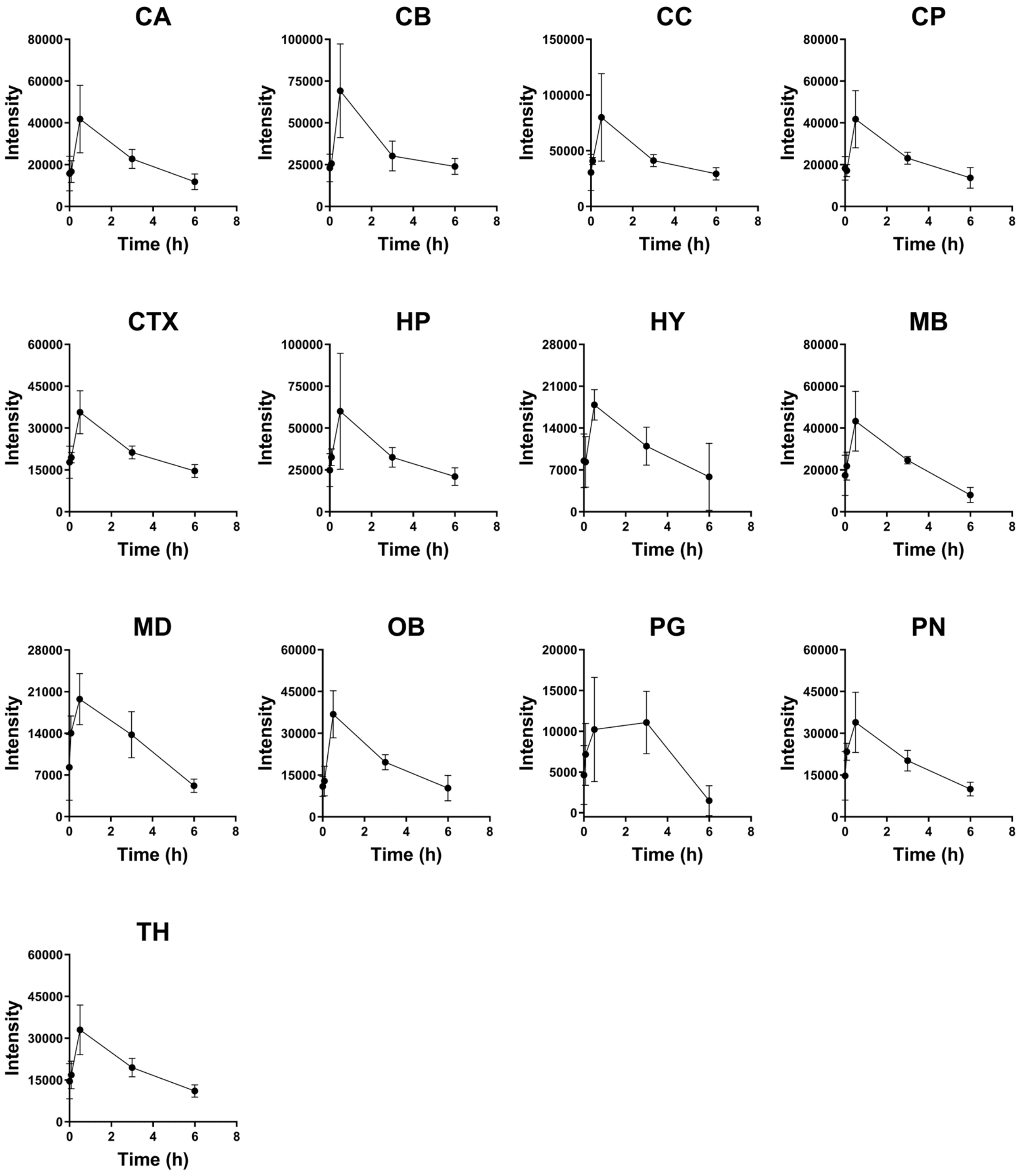

Figure S11 The relative intensity changes of Adenosine in the different brain microregions with the time. Data are presented as means ± standard deviation (SD), $n = 3$.

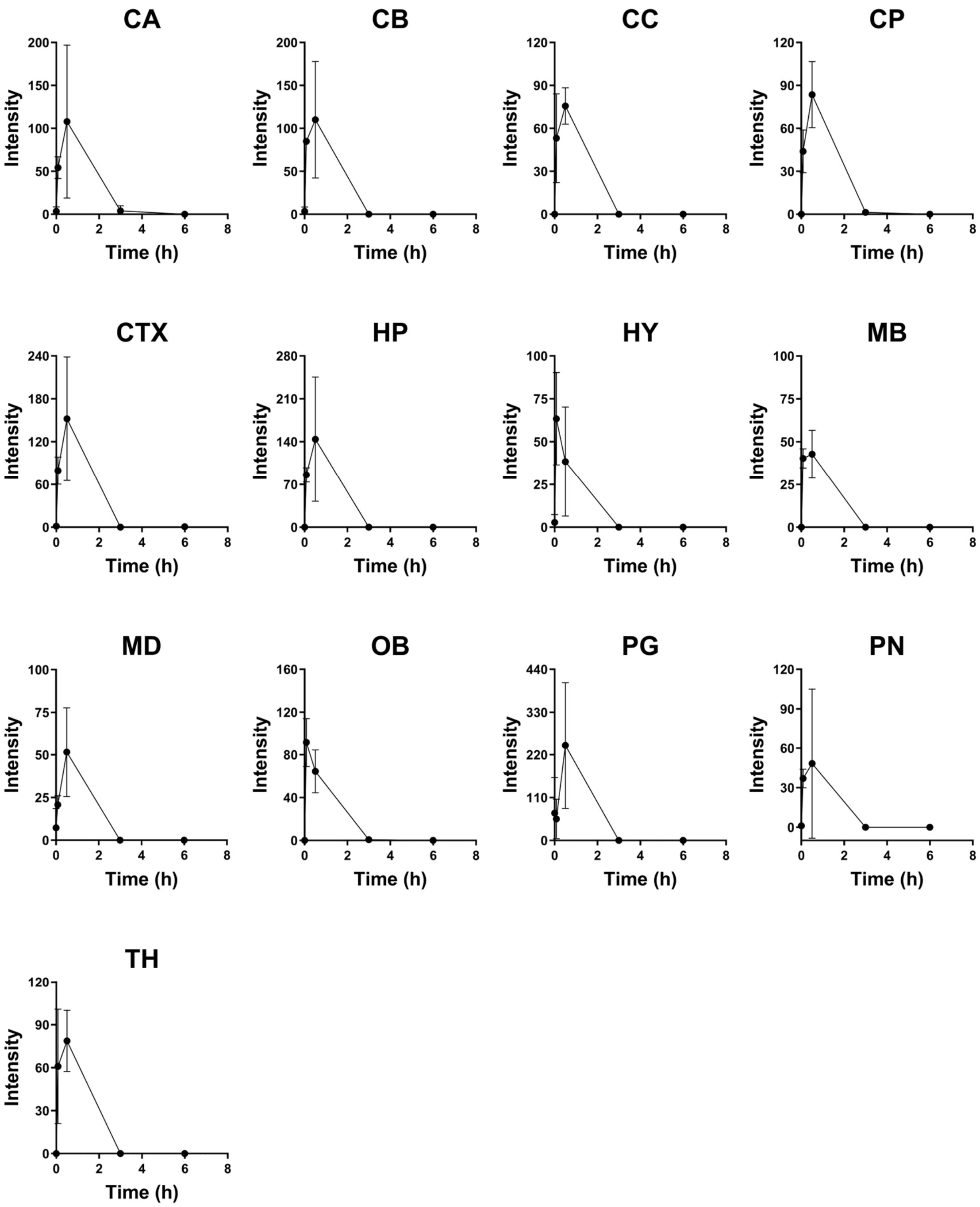

Figure S12 The relative intensity changes of melatonin in the different brain microregions with the time. Data are presented as means ± standard deviation (SD), $n = 3$.

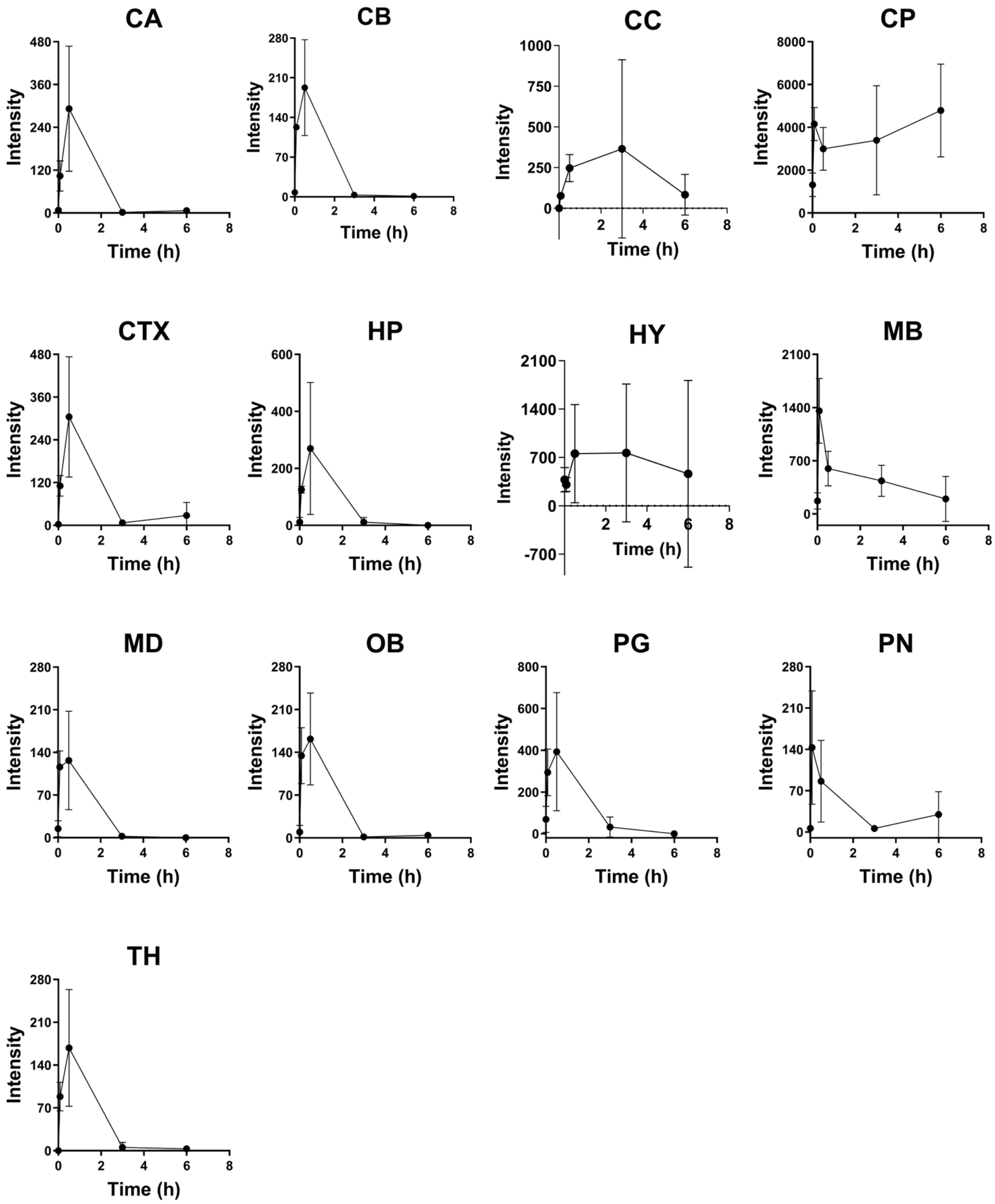

Figure S13 The relative intensity changes of DA in the different brain microregions with the time. Data are presented as means ± standard deviation (SD), $n = 3$.

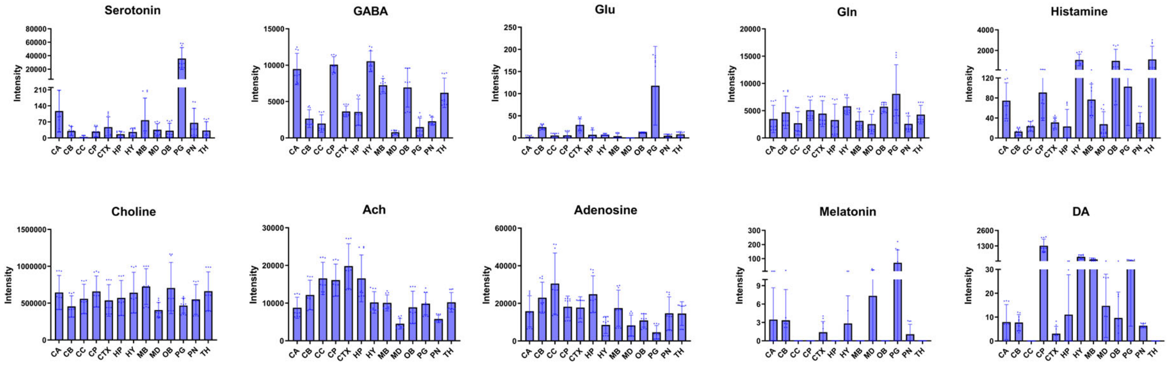

Figure S14 Relative intensity of NTs in each brain microregion before administration of 1,8-Cineole to rats.

Table S2 The detailed information of the detected metabolites by AFADESI-MSI in positive ion mode.

Metabolite	Formula	Ion type	Measured *m/z*	Theoretical *m/z*	Relative error (ppm)	MS^2
1,8-Cineole	$C_{10}H_{18}O$	$[M+H]^+$	155.1432	155.1430	0.99	137.1326、81.0700
9-hydroxy-1,8-Cineole	$C_{10}H_{18}O_2$	$[M+H]^+$	171.1381	171.1380	0.84	
Cineolic acids	$C_{10}H_{16}O_5$	$[M+H]^+$	217.1071	217.1070	0.25	
Tryptophan	$C_{11}H_{12}N_2O_2$	$[M+H]^+$	205.0973	205.0972	0.70	
5-hydroxytryptophan	$C_{11}H_{12}N_2O_3$	$[M+H]^+$	221.0939	221.0921	8.29	
Serotonin	$C_{10}H_{12}N_2O$	$[M+H]^+$	177.1024	177.1022	0.92	
5-Hydroxyindoleacetaldehyde	$C_{10}H_9NO_2$	$[M+H]^+$	176.0708	176.0706	1.10	
5-Hydroxyindoleacetic acid	$C_{10}H_9NO_3$	$[M+H]^+$	192.0656	192.0655	0.43	
Melatonin	$C_{13}H_{16}N_2O_2$	$[M+H]^+$	233.1286	233.1285	0.62	
N-acetyl-5-hydroxytryptamine	$C_{12}H_{14}N_2O_2$	$[M+H]^+$	219.1131	219.1128	1.34	
Glutamine	$C_5H_{10}N_2O_3$	$[M+H]^+$	147.0766	147.0764	1.25	84.0440、101.0706、130.0492
Glutamate	$C_5H_9NO_4$	$[M+H]^+$	148.0606	148.0604	1.10	
gamma-Aminobutyric acid	$C_4H_9NO_2$	$[M+H]^+$	104.0708	104.0706	1.86	68.0493、69.0332、86.0597、87.0437
Succinic acid	$C_4H_6O_4$	$[M+H]^+$	119.0339	119.0339	0.11	
Histidine	$C_6H_9N_3O_2$	$[M+H]^+$	156.0769	156.0768	0.92	95.0599、110.0710

Histamine	$C_5H_9N_3$	$[M+H]^+$	112.0871	112.0869	1.55	
N-methylhistamine	$C_6H_{11}N_3$	$[M+H]^+$	126.1027	126.1026	0.98	
Tyrosine	$C_9H_{11}NO_3$	$[M+H]^+$	182.0813	182.0812	0.73	
Dopa	$C_9H_{11}NO_4$	$[M+H]^+$	198.0764	198.0761	1.58	
Dopamine	$C_8H_{11}NO_2$	$[M+H]^+$	154.0864	154.0863	0.93	
3,4-Dihydroxyphenylacetaldehyde	$C_8H_8O_3$	$[M+H]^+$	153.0547	153.0546	0.54	
3-Methoxytyramine	$C_9H_{13}NO_2$	$[M+H]^+$	168.1021	168.1019	1.15	
NorEpinephrine (NE)	$C_8H_{11}NO_3$	$[M+H]^+$	170.0813	170.0812	0.78	
3，4-Dihydroxymandelicacid	$C_8H_8O_5$	$[M+H]^+$	185.0445	185.0444	0.29	
Acetylcholine	$C_7H_{16}NO_2^+$	$[M+H]^+$	146.1177	146.1176	0.68	
ADP	$C_{10}H_{15}N_5O_{10}P_2$	$[M+H]^+$	428.0367	428.0367	0.01	
ATP	$C_{10}H_{16}N_5O_{13}P_3$	$[M+H]^+$	508.0006	508.0030	-4.78	
Adenosine	$C_{10}H_{13}N_5O_4$	$[M+H]^+$	268.1041	268.1040	0.27	
Homocysteine	$C_4H_9NO_2S$	$[M+H]^+$	136.0429	136.0427	1.64	
Betaine	$C_5H_{11}NO_2$	$[M+H]^+$	118.0864	118.0863	1.21	59.0728
Spermine	$C_{10}H_{26}N_4$	$[M+H]^+$	203.2231	203.2230	0.36	
Docosahexaenoic acid	$C_{22}H_{32}O_2$	$[M+H]^+$	329.2475	329.2475	-0.02	
Adenine	$C_5H_5N_5$	$[M+H]^+$	136.0619	136.0618	0.91	
Taurine	$C_2H_7NO_3S$	$[M+H]^+$	126.0221	126.0219	1.30	96.9951、108.0110
Creatine	$C_4H_9N_3O_2$	$[M+H]^+$	132.0769	132.0768	1.09	
Proline	$C_5H_9NO_2$	$[M+H]^+$	116.0708	116.0706	1.67	
Inosine	$C_{10}H_{12}N_4O_5$	$[M+H]^+$	269.0881	269.0880	0.20	
Asparagine	$C_4H_8N_2O_3$	$[M+H]^+$	133.0609	133.0608	1.00	
3,4-Dihydroxymandelic acid	$C_8H_8O_5$	$[M+H]^+$	185.0445	185.0444	0.29	
Choline	$C_5H_{14}NO$	$[M+H]^+$	104.1071	104.1070	0.96	58.0651、60.0806、87.0439

Table S3 Differential Metabolites Detected by Mass Spectrometry Imaging Targeting the Tryptophan-Serotonin-Melatonin (Try-5-HT-Mel) and Glutamine-Glutamate-γ-Aminobutyric Acid (Gln-Glu-GABA) Metabolic Pathways.

Metabolite	Brain regions	Control	Treatment	Adjusted P Value

Tryptophan	CA	3.88±2.92	137.11±91.78****	<0.0001
	CB	6.54±6.06	132.81±46.72****	<0.0001
	CC	0±0	130.27±101.16****	<0.0001
	CP	0±0	109.98±24.36****	<0.0001
	CTX	8.53±3.69	145.38±74.19****	<0.0001
	HP	7.84±11.77	130.55±61.27****	<0.0001
	HY	5.69±4.94	117.58±26.86****	<0.0001
	MB	0±0	85.29±23.38****	<0.0001
	MD	14.63±17.96	63.91±15.55	0.0811
	OB	3.81±4.31	109.44±20.69****	<0.0001
	PG	55.85±83.8	160.12±190.56****	<0.0001
	PN	4.84±1.75	63.54±39.79*	0.0167
	TH	4.22±6.33	106.82±64.63****	<0.0001
5-HTP	CA	964.5±1239.8****	579.33±204.53	<0.0001
	CB	936.41±1223.87***	616.48±164.57	0.0004
	CC	968.26±1215.11	828.43±108.38	0.546
	CP	1059.85±1224.94****	608.08±138.28	<0.0001
	CTX	1067.28±1358.13****	482.66±73.97	<0.0001
	HP	839.74±1040.55****	427.72±49.03	<0.0001
	HY	891.32±985.98***	569.61±78.08	0.0004
	MB	859.48±963.53	940.22±208.14	0.984
	MD	864.1±1152.85****	511.92±165.48	<0.0001
	OB	812.47±882.21*	558.79±54.9	0.0102
	PG	992.06±1321.82****	315.7±248.23	<0.0001
	PN	852.29±1012.27	780.98±364.9	0.9947
	TH	926.77±1133.21	799.61±151.3	0.6866
Serotonin	CA	117.27±90.92	778.42±565.44	0.0567
	CB	30.27±19.84	519.28±358.19*	0.0461
	CC	4.07±5.69	399.19±295.06*	0.0471
	CP	27.36±22.25	511.46±611.66	0.4142
	CTX	46.81±45.4	919.25±617.87*	0.0329
	HP	16.23±13.16	740.27±518.63*	0.0405
	HY	25.9±17.54	561.11±729.43	0.5326
	MB	77.44±96.26	705.9±852.93	0.3974
	MD	35.79±26.82	271.16±360.32	0.6008
	OB	31.39±32.73	338.79±319.26	0.1595
	PG	35890.43±16263.61	36736.5±14876.74	>0.9999

	PN	66.17±62.97	253.35±381.09	0.9485
	TH	32.45±38.24	447.95±439.26	0.1821
	CA	2.35±3.53	159.93±123.27****	<0.0001
	CB	3.78±3.1	135.97±80.93****	<0.0001
	CC	0±0	148.75±95.53****	<0.0001
	CP	0.6±1.81	102.43±43.59**	0.0011
	CTX	9.52±2.94	178.16±147.25****	<0.0001
	HP	0±0	172.87±107.93****	<0.0001
N-ace-5-HTP	HY	2.18±3.81	118.68±37.52***	0.0001
	MB	3.04±4.56	64.94±26.54	0.1674
	MD	5.82±6.81	68.88±56.84	0.1491
	OB	3.42±5.81	105.03±31.24**	0.0011
	PG	4.36±13.1	106.04±160.86**	0.0011
	PN	3.56±2.82	45.6±36.21	0.716
	TH	2.35±3.53	84.31±79.56*	0.0167
	CA	3.46±5.19	107.9±89.03****	<0.0001
	CB	3.33±5.04	110.03±67.85****	<0.0001
	CC	0±0	75.63±12.74***	0.0005
	CP	0±0	83.51±23.17****	<0.0001
	CTX	1.4±1.75	152.18±86.44****	<0.0001
	HP	0±0	144.18±101.56****	<0.0001
Melatonin	HY	2.82±4.51	38.35±31.8	0.447
	MB	0±0	42.76±13.94	0.1902
	MD	7.33±11.23	51.6±26.11	0.1543
	OB	0±0	64.53±20.09**	0.0047
	PG	61.72±93.87	243.87±161.92****	<0.0001
	PN	1.07±1.61	48.36±56.66	0.099
	TH	0±0	78.74±21.44***	0.0002
	CA	0±0	5.04±3.86	0.7479
	CB	1.48±1.11	3.47±2.79	>0.9999
	CC	0±0	14.26±14.06***	0.0001
	CP	0±0	9.48±3.11*	0.0312
5-HIAA	CTX	0±0	10.66±4.9**	0.0091
	HP	0±0	0±0	>0.9999
	HY	0±0	4.62±6.94	0.8405
	MB	0±0	9.05±5.14*	0.047
	MD	0±0	2.19±4.64	0.9998

	OB	0±0	7.74±3.75	0.1518
	PG	0±0	17.27±25.91****	<0.0001
	PN	1.33±2	5.13±4.67	0.9568
	TH	0±0	6.29±4.75	0.4223
Gln	CA	3472.74±2520.9	14195.1±4001.18****	<0.0001
	CB	4695.24±2968.34	16549.44±4729.13****	<0.0001
	CC	2714.16±2150.54	11819.09±3044.14****	<0.0001
	CP	5090.21±1872.63	15938.74±3277.16****	<0.0001
	CTX	4469.72±2378.42	15548.84±3532.18****	<0.0001
	HP	3283.31±2925.92	13681.43±2787.61****	<0.0001
	HY	5820.5±1551.67	16939.55±5454.91****	<0.0001
	MB	3142.67±1673.78	11612.78±3842****	<0.0001
	MD	2543.95±1791.25	7370.37±1669.6***	0.0005
	OB	5717.76±863.09	15427.87±2681****	<0.0001
	PG	8089.38±5357.86	26628.87±14278.46****	<0.0001
	PN	2583.77±1464.86	7636.43±3422.63***	0.0002
	TH	4296.79±1702.79	14784.43±4502.38****	<0.0001
Glu	CA	2.5±3	831.05±607.45	0.2865
	CB	24.44±5.68	1570.59±955.49***	0.0007
	CC	5.52±5.28	654.56±440.42	0.6566
	CP	5.94±7.93	706.67±630.28	0.5412
	CTX	29.37±13.84	2747.92±1773.05****	<0.0001
	HP	7.2±10.85	1304.9±909.55**	0.0077
	HY	7.52±2.06	765.02±917.77	0.4188
	MB	3.99±5.99	307.24±303.02	0.9989
	MD	0±0	126.08±64.76	>0.9999
	OB	12.61±1.22	404.71±237.05	0.9875
	PG	117.97±88.82	4353.13±3778.66****	<0.0001
	PN	5.19±3.93	120.58±117.38	>0.9999
	TH	8.3±6.28	1433.93±1221.32**	0.0023
GABA	CA	9465.91±2147.12	16300.99±2822.99****	<0.0001
	CB	2658±1217.02	6727.17±1330.38****	<0.0001
	CC	1986.46±1197.37	5218.49±1609.6****	<0.0001
	CP	10071.02±1091.67	17623.58±4531.57****	<0.0001
	CTX	3646.55±745.42	7278.01±1115.28****	<0.0001
	HP	3574.05±1790.9	8265.4±1502.64****	<0.0001
	HY	10526.54±1430.45	20022.27±1730.81****	<0.0001

	MB	7248.55±919.6	14796.74±2814.13****	<0.0001
	MD	777.73±292.86	3544.61±1316.23****	<0.0001
	OB	6925.54±2677.59	17734.82±1082.23****	<0.0001
	PG	1493.02±1251.43	3206.26±1630.72*	0.0385
	PN	2290.54±558.34	5746.55±870.64****	<0.0001
	TH	6207.39±2025.68	10599.56±2489.98****	<0.0001
Succinic acid	CA	0±0	1.08±2.15	0.9991
	CB	1.69±1.85	0.94±0.7	>0.9999
	CC	0±0	5.48±8.23**	0.001
	CP	0±0	0±0	>0.9999
	CTX	0.63±0.94	2.95±1.42	0.6773
	HP	0±0	0±0	>0.9999
	HY	2.19±2.09	2.91±4.37	>0.9999
	MB	1.51±2.27	2.6±1.96	0.999
	MD	2.49±3.75	2.85±5.67	>0.9999
	OB	2.01±1.5	0±0	0.8451
	PG	0±0	0±0	>0.9999
	PN	0±0	0.59±1.18	>0.9999
	TH	0±0	2.48±3.73	0.5773

The data are presented as the mean ± SD (standard deviation). * $P < 0.05$, ** $P < 0.01$, *** $P < 0.001$, **** $P < 0.0001$ versus untreated control group. PG: pineal gland, CC: corpus callosum, HP: hippocampus, CB: cerebellar, CA: cerebral aqueduct, CTX: cerebral cortex, MB: middle brain, TH: thalamus, MD: medulla, PN: pons; HY: hypothalamus, CP: caudate putamen, OB: olfactory bulb.

Table S4 Spatial Distribution and Alterations of 5-HT and GABA Pathway Metabolites in Rat Brain 30 min Post 1,8-Cineole Treatment Compared to Control.

Metabolite	Microregion	Relative Abundance (Control)%	Relative Abundance (Treatment)%	FC	Log2（FC）
**Tryptophan**	CA	4	9	4.8224	28.2927
	CB	6	9	4.1483	17.7321
	CC	1	9	7.0364	131.2707
	CP	1	7	6.7943	110.9886
	CTX	7	10	3.9397	15.3448

	HP	7	9	3.8951	14.8784
	HY	5	8	4.1463	17.7072
	MB	1	6	6.4312	86.2936
	MD	12	4	2.0540	4.1526
	OB	4	7	4.5195	22.9356
	PG	44	11	1.5027	2.8338
	PN	5	4	3.4645	11.0386
	TH	4	7	4.3682	20.6512
**5-HTP**	CA	8	7	-0.7344	0.6011
	CB	8	8	-0.6023	0.6587
	CC	8	10	-0.2248	0.8557
	CP	9	8	-0.8005	0.5741
	CTX	9	6	-1.1432	0.4528
	HP	7	5	-0.9716	0.5099
	HY	7	7	-0.6451	0.6395
	MB	7	12	0.1294	1.0938
	MD	7	6	-0.7541	0.5929
	OB	7	7	-0.5392	0.6882
	PG	8	4	-1.6488	0.3189
	PN	7	10	-0.1259	0.9164
	TH	8	10	-0.2127	0.8629
**Serotonin**	CA	0	2	2.7202	6.5898
	CB	0	1	4.0562	16.6352
	CC	0	1	6.3006	78.8234
	CP	0	1	4.1751	18.0644
	CTX	0	2	4.2664	19.2445
	HP	0	2	5.4263	43.0016
	HY	0	1	4.3849	20.8919
	MB	0	2	3.1717	9.0109
	MD	0	1	2.8869	7.3968
	OB	0	1	3.3910	10.4904
	PG	99	85	0.0336	1.0236
	PN	0	1	1.9207	3.7862
	TH	0	1	3.7462	13.4194
**N-ace-5-HTP**	CA	6	11	5.5841	47.9706
	CB	9	9	4.8397	28.6340
	CC	2	10	7.2264	149.7514

	CP	3	7	6.0106	64.4737
	CTX	19	12	4.0895	17.0237
	HP	2	12	7.4419	173.8783
	HY	6	8	5.2322	37.5880
	MB	7	4	4.0274	16.3072
	MD	13	5	3.3563	10.2414
	OB	8	7	4.5827	23.9629
	PG	10	7	4.3176	19.9402
	PN	8	3	3.3534	10.2207
	TH	6	6	4.6673	25.4103
**Melatonin**	CA	5	9	4.6094	24.4103
	CB	5	9	4.6790	25.6161
	CC	1	6	6.2598	76.6304
	CP	1	7	6.4012	84.5198
	CTX	3	12	5.9954	63.7982
	HP	1	12	7.1818	145.1857
	HY	4	3	3.3616	10.2785
	MB	1	3	5.4517	43.7642
	MD	9	4	2.6587	6.3146
	OB	1	5	6.0342	65.5331
	PG	67	20	1.9648	3.9037
	PN	2	4	4.5710	23.7690
	TH	1	6	6.3173	79.7417
**5-HIAA**	CA	6	6	2.5965	6.0480
	CB	16	4	0.8487	1.8009
	CC	6	14	3.9322	15.2652
	CP	6	10	3.3897	10.4808
	CTX	6	11	3.5441	11.6653
	HP	6	1	0.0000	1.0000
	HY	6	5	2.4929	5.6289
	MB	6	9	3.3305	10.0596
	MD	6	3	1.6752	3.1936
	OB	6	8	3.1280	8.7425
	PG	6	17	4.1918	18.2745
	PN	15	6	1.3933	2.6267
	TH	6	7	2.8671	7.2962
**Gln**	CA	6	8	2.0309	4.0867

	CB	8	9	1.8173	3.5242
	CC	5	6	2.1221	4.3534
	CP	9	8	1.6465	3.1308
	CTX	8	8	1.7983	3.4781
	HP	6	7	2.0587	4.1660
	HY	10	9	1.5410	2.9100
	MB	6	6	1.8853	3.6943
	MD	5	4	1.5343	2.8965
	OB	10	8	1.4319	2.6979
	PG	14	14	1.7188	3.2915
	PN	5	4	1.5631	2.9548
	TH	8	8	1.7825	3.4402
**Glu**	CA	1	5	7.8892	237.0672
	CB	10	10	5.9487	61.7633
	CC	3	4	6.6500	100.4243
	CP	3	5	6.6701	101.8387
	CTX	12	18	6.4998	90.4974
	HP	3	9	7.3139	159.1162
	HY	3	5	6.4895	89.8538
	MB	2	2	5.9478	61.7257
	MD	0	1	6.9896	127.0802
	OB	6	3	4.8968	29.7919
	PG	49	28	5.1937	36.5983
	PN	3	1	4.2946	19.6242
	TH	4	9	7.2688	154.2133
**GABA**	CA	14	12	0.7841	1.7220
	CB	4	5	1.3393	2.5303
	CC	3	4	1.3930	2.6262
	CP	15	13	0.8072	1.7499
	CTX	5	5	0.9968	1.9956
	HP	5	6	1.2093	2.3122
	HY	16	15	0.9275	1.9020
	MB	11	11	1.0294	2.0412
	MD	1	3	2.1868	4.5531
	OB	10	13	1.3565	2.5606
	PG	2	2	1.1021	2.1467
	PN	3	4	1.3266	2.5082

	TH	9	8	0.7718	1.7075
**Succinic acid**	CA	4	6	1.0624	2.0884
	CB	11	6	-0.4702	0.7219
	CC	4	19	2.6967	6.4831
	CP	4	3	0.0000	1.0000
	CTX	7	11	1.2774	2.4240
	HP	4	3	0.0000	1.0000
	HY	14	11	0.2937	1.2257
	MB	11	10	0.5202	1.4342
	MD	15	11	0.1415	1.1031
	OB	13	3	-1.5900	0.3322
	PG	4	3	0.0000	1.0000
	PN	4	5	0.6763	1.5980
	TH	4	10	1.8030	3.4896

A Log2(FC) value of 0 (corresponding to an FC of 1) indicates no change in metabolite levels between the treatment and control groups. An FC > 1 signifies up-regulation of the metabolite signal in the treatment group, while an FC < 1 signifies down-regulation of the signal intensity in the treatment group. PG: pineal gland, CC: corpus callosum, HP: hippocampus, CB: cerebellar, CA: cerebral aqueduct, CTX: cerebral cortex, MB: middle brain, TH: thalamus, MD: medulla, PN: pons; HY: hypothalamus, CP: caudate putamen, OB: olfactory bulb.
